# Supplementary material for: Safety assessment of multiple systemic administration of human mesenchymal stem cell-conditioned medium for various chronic diseases
Source: PLoS One. 2025 May 6;20(5):e0322497. doi: 10.1371/journal.pone.0322497 (PMC12054860; doi:10.1371/journal.pone.0322497)
Supplement: Attachment — Submitted filename: Review report-12.08.2024.docx [file pone.0322497.s001.docx]

The mesenchymal stem cell-conditioned medium gives promising results in in vitro and in vivo studies, and it is exciting to see that these mediums can be applied to humans. Therefore, the presented work is valuable, but some requirements must be met in order to be publishable.

Introduction

1. The introduction is weak for this study. The importance of using the conditioned medium derived from mesenchymal stem cells for inflammatory diseases should be emphasized. The literature contains many in vitro and in vivo studies on this subject. The introduction should be supported by these studies. Why is it desired to be used in humans? This context should be well established.

Methods

2. How many donors were used as sources of mesenchymal stem cells? Was CM prepared separately from the cells obtained from these donors? Which source was the CM used for which disease? It should be specified.
3. Has the content of CM obtained from adipose tissue been compared with that of CM obtained from the umbilical cord? This difference is significant, and the study should have been planned with this in mind.
4. The preparation conditions of CMs should be explained in the method section, such as the number of cells, how long they were kept, how much medium, medium, and supplement content were used, etc.
5. How were the doses determined? Your sample size for INH is 1. This is insufficient.

Results

6. What do 1 and 2 represent in Figure 2? The x-axis naming of all graphs should be corrected. Label in an understandable way when looking at the graph or add a description to the figure legend. It is irrational for the "error bar SE" label to be on the x-axis. Add this to the figure legend and label it in a way that makes your data understandable.

Discussion

7. The findings should be supported by literature data in the discussion. Possible safety and side effects should be discussed with possible reasons, considering variables such as administration routes, CM source used, disease, etc.
